# Wealth, health, cats and *Toxoplasma gondii*: An ecological correlational study of subjective well-being across 93 countries

**DOI:** 10.1371/journal.pone.0357911

**Published:** 2026-09-30

**Authors:** Javier I. Borráz-León, Markus J. Rantala, Indrikis Krams, Dario Maestripieri

**Affiliations:** 1 Secretariat of Science, Humanities, Technology, and Innovation, Mexico City, Mexico; 2 Behavioral Biology Lab, National Institute of Psychiatry “Ramón de la Fuente Muñiz”, Mexico City, Mexico; 3 Department of Biology, University of Turku, Turku, Finland; 4 National Institute of Research and Innovation, Riga, Latvia; 5 Department of Ecology, Faculty of Medicine and Life Sciences, University of Latvia, Riga, Latvia; 6 Department of Biodiversity, Institute of Life Sciences and Technology, Daugavpils University, Daugavpils, Latvia; 7 Institute for Mind and Biology, The University of Chicago, Chicago, Illinois, United States of America; 8 Department of Comparative Human Development, The University of Chicago, Chicago, Illinois, United States of America; De Montfort University Faculty of Health and Life Sciences, UNITED KINGDOM OF GREAT BRITAIN AND NORTHERN IRELAND

## Abstract

National happiness is related not only to socioeconomic factors but also to biological and ecological factors. This ecological correlational study examined associations between national happiness, cat density, *Toxoplasma gondii* exposure, and socioeconomic indicators across 93 countries. We hypothesized that countries with higher socioeconomic indicators (e.g., GDP, health expenditure, income level) would exhibit higher happiness levels. In contrast, we predicted that higher *T. gondii* exposure, both acute (IgM) and chronic (IgG), would be negatively associated with national happiness. Data on happiness measures, cat density, GDP per capita, income classification, health expenditure, and *T. gondii* seroprevalence in pregnant women (as a proxy for broader population exposure) were obtained from publicly available sources. In line with our hypothesis, we found that national happiness was positively associated with cat density and some socioeconomic factors and negatively associated with IgM seroprevalence. In multivariate analyses, cats per 10,000 inhabitants and IgM seroprevalence remained the strongest independent predictors. Overall, these exploratory analyses suggest that biological and ecological factors are associated with socioeconomic variables in determining national well-being. Future studies of happiness at the national level should integrate both socioeconomic and biological perspectives and include longitudinal and interdisciplinary approaches to elucidate the underlying mechanisms and processes.

## Introduction

Subjective well-being, commonly assessed through life satisfaction and happiness indicators, has increased its relevance as a central indicator of human and social development [1,2]. While some economic indicators such as gross domestic product (GDP) per capita, family income, or health expenditure are known to be positively associated with well-being [e.g., 3,4], they are not sufficient to completely explain the variability of happiness levels across countries [5–8]. For example, previous research has shown that loneliness is linked to low happiness, especially among individuals who are single, divorced, or without children, or among aging individuals who have lost family members or other sources of social support [9–13]. The prevalence of loneliness in a particular population, society, or country, in turn, has been weakly associated with socioeconomic indicators [10]. This has led to a growing interest in understanding how socioeconomic indicators interact with psychosocial factors to influence subjective well-being on a population scale [e.g., 7,10,12,14,15].

Companion animal ownership has been proposed to be an important contributor to psychological health [16–18]. For example, previous studies have suggested that the presence of pets, particularly cats and dogs, can reduce loneliness, foster emotional bonding, diminish stress, and improve mood [16–21]. Moreover, pets are often regarded as family members in many societies, and their care reflects cultural values related to empathy, leisure, and relational well-being [22]. Thus, the number of companion animals within a society or country might serve as a sociobehavioral indicator of lifestyle quality and social norms related to animals.

Pet ownership, however, especially cats, also entails some health risks. For instance, domestic cats (*Felis catus*) are the definitive hosts of the neuroparasite *Toxoplasma gondii* (*T. gondii*), a globally widespread protozoan capable of infecting nearly all warm-blooded animals, including humans [23–26]. Globally, approximately 10–30% of adults in any given human population are estimated to be infected with *T. gondii*, often without even knowing it [25,27–29]. Human infection occurs mainly through ingestion of oocysts excreted in cat feces, consumption of undercooked contaminated meat, and potentially, sexual intercourse with an infected person [23,30–32, see 33 for a review]. In this regard, a growing body of research has suggested that latent *Toxoplasma* infection may be associated with the development of psychopathological conditions such as schizophrenia, obsessive-compulsive disorder, autism spectrum disorders, anxiety disorders, depression, and personality disorders [25,30,34–37], either through direct effects of infection or through mediational pathways associated with chronic stress, neurotransmitter dysregulations, and chronic neuroinflammation [37,38].

Most *T. gondii* infections are known to be clinically asymptomatic in healthy individuals; however, serological markers may help distinguish between recent and past exposures. Whereas IgM antibodies indicate acute or recent infections associated with inflammation that could affect mood and cognition, IgG antibodies signal latent or chronic infections that have been linked to various psychopathologies [30,39–41]. The fact that *T. gondii* can have adverse effects on cognition, physiology, behavior and emotional regulation on a large scale [e.g., 26,31,33,42] raises important questions about its potential role in modulating aggregate measures such as national happiness [43,44].

The idea that domestic cats may provide psychological and emotional benefits to their owners but also serve as a vector for pathogens with negative mental health outcomes, represents a compelling biopsychological paradox, with implications for well-being and general health in different countries depending on factors such as disparities in sanitation infrastructure, pet management, and public health systems [45,46]. It is reasonable to assume that in wealthier countries, cats are more likely to be kept indoors, vaccinated, and fed industrial pet food, minimizing opportunities for environmental contamination and zoonotic transmission [47], whereas in low- and middle-income countries, free-roaming cats might represent a greater risk for community-level parasite exposure [48]. Thus, the ecological impact of cats on human well-being may depend not only on their presence, but also on the conditions under which humans and non-human animals interact; conditions that are shaped by national indicators such as wealth, policy, and culture [e.g., 47,49,50].

Despite the intuitive connections among these variables, to our knowledge, no prior study has simultaneously examined the associations between relative cat population, *T. gondii* exposure, economic indicators, and national happiness across a large set of countries. To address this gap, the present study aimed to explore how cats per 10,000 inhabitants (cat density), *T. gondii* IgM and IgG seroprevalence, GDP, income classification, and health expenditure are related to national happiness across 93 countries. This ecological correlational framework offers a transdisciplinary approach that integrates psychosocial, parasitological, economic, and public health indicators to explore how biological and ecological risks may be amplified or buffered by socioeconomic factors.

The primary hypothesis tested in this study is that countries with higher economic indicators (i.e., GDP, health expenditure, income level) will exhibit higher levels of happiness. In contrast, we predict that greater *T. gondii* seroprevalence, both chronic (IgG) and recent (IgM), will be negatively associated with national happiness. As an exploratory analysis, we further hypothesize that cat density may be positively associated with national happiness, but that this association is weakened or even reversed through the mediating effect of *T. gondii* seroprevalence, suggesting that parasite infection may be as important as economic factors in shaping how the presence of cats may relate to national well-being. By addressing this constellation of variables across countries, this study aims to provide new conceptual and empirical insights into how socioeconomic indicators, pets, and parasitic exposure interact to influence global patterns of health and happiness.

## Methods

### Data compilation

Data on national happiness levels, cat density, socioeconomic indicators, and *T. gondii* seroprevalence, both IgG and IgM, were compiled for 93 countries (Fig 1) using publicly available sources. Data sources and temporal coverage of the study variables are summarized in Table 1. The final number of countries was determined based on the availability of complete data across all variables of interest, ensuring comparability throughout the analysis. Based on a G*Power post-hoc analysis, our sample size (*N* = 93 and *α* = 0.05) had 85% power to detect moderate effect sizes (*r* = 0.3). However, the present study was underpowered to reliably detect small effects (e.g., *r* < 0.20). Therefore, the non-significant associations should be interpreted cautiously, as the absence of statistical significance does not necessarily indicate the absence of a true association.

**Table 1:** Data sources and temporal coverage of the study variables.

Variable	Source	Time period
Happiness scores	World Population Review (based on World Happiness Report / Gallup World Poll)	3-year average: 2022–2024
Cat density (cats per 10,000 inhabitants)	Cat Population by Country 2025 dataset (worldstats.com/World Population Review)	2025 estimates (projections based on compilation data)
GDP per capita (current US$)	World Bank database	Most recent available year (typically 2023–2024, depending on country reporting)
Health expenditure (% of GDP)	WHO Global Health Expenditure Database	Primarily 2021–2023 (latest update includes 2023 data)
Country income classification	World Bank 2025 classifications	Based on GNI per capita for 2023 or 2024 (FY2025/FY2026 classifications)
*T. gondii* IgM and IgG seroprevalence (in pregnant women)	Bigna et al. (2020)	Individual studies: 1976–2017 (published 1979–2018); meta-analysis published 2020

*Note*: GDP = Gross Domestic Product (in current $US Dollars), IgM and IgG = Immunoglobulin M and G, respectively.

**Fig 1:**
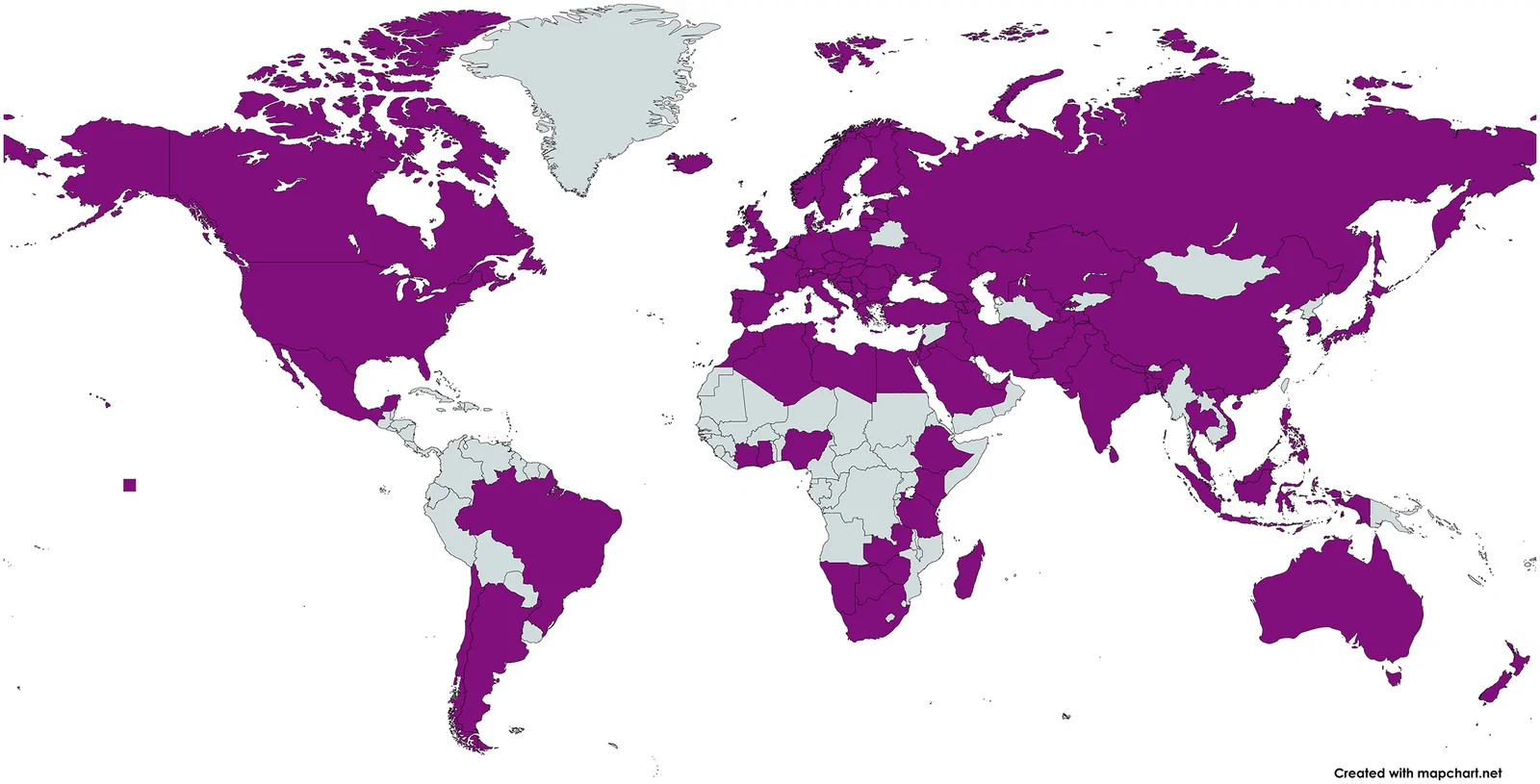
Countries shown in purple represent those included in the study. *Note:* Created with mapchart.net under a CC BY 4.0 License. A complete list of the countries can be found in Supplementary Materials.

### Happiness index

Happiness scores were obtained from the World Population Review website (https://worldpopulationreview.com/country-rankings/happiest-countries-in-the-world), based on their 2025 report of the Happiest Countries in the World. These rankings reflect three-year averages (i.e., 2022–2024) and are determined by analyzing comprehensive Gallup polling data from 149 countries in six particular categories: GDP per capita, social support, healthy life expectancy, freedom to make your own life choices, generosity of the general population, and perceptions of internal and external corruption levels (see the World Population Review website for more details). For the purposes of this study, we used data from the 93 countries for which complete information on all other variables (see below) was available.

### Cat density

Estimates of domestic cat populations per country were obtained from the Cat Population By Country 2025 dataset (https://worldstats.com/country-stats/cat-population-by-country). To allow cross-national comparisons, the number of domestic cats per country was standardized by population size. Thus, we calculated the number of cats per 10,000 inhabitants by dividing the total number of cats in each country by the total human population (sourced from: https://data.worldbank.org/indicators/SP.POP.TOTL), and then multiplying the result by 10,000. This standardization allows for more interpretable ecological comparisons for differences in population size across countries [51].

### Gross Domestic Product

Gross Domestic Product per capita (in US dollars) was retrieved from the World Bank 2025 database (https://data.worldbank.org/indicator/NY.GDP.PCAP.CD). This measure indicates average economic productivity per person and is often used as a marker of national wealth.

### Health expenditure

Data on current health expenditure (as percentage of GDP) were obtained from the World Health Organization’s Global Health Expenditure Database (https://apps.who.int/gho/data/view.main.GHEDCHEGDPSHA2011v?lang=en), updated in 2023. This measure indicates the total health-related spending, both public and private, excluding water and sanitation. Reporting expenditure as a percentage of GDP allows for comparisons of national health priorities relative to economic output and is frequently used in global health research [52].

### Country income classification

Country income groups were classified according to the World Bank’s 2025 income classifications (https://datahelpdesk.worldbank.org/knowlegebase/articles/906519), which are based on gross national income per capita using the Atlas method. Accordingly, countries were categorized as 1 = low, 2 = lower-middle, 3 = upper-middle, or 4 = high income.

### IgM and IgG seroprevalence

Due to the scarcity of comprehensive *T. gondii* seroprevalence data for the general population across the 93 countries included in this study, we employed the seroprevalence data from pregnant women reported in Bigna et al. [28] as a proxy for community-level exposure. The use of these specific categories ensures methodological alignment with international benchmarks, facilitating a direct comparison with the most recent peer-validated epidemiological models. This approach is supported by prior research showing that seroprevalence rates in pregnant women closely reflect broader population exposure levels [e.g., 24,27], a correlation also noted by Bigna et al. [28]. While this approach might introduce a demographic bias by focusing only on pregnant women, the routine screening of this population for *T. gondii* due to congenital transmission provides a robust and standardized dataset for cross-national comparisons [28]. See the Limitations section for further discussion and implications of the use of this proxy.

As reported in Bigna et al. [28], national IgG seroprevalence was categorized into five levels as follows: 1 = <10.0%, 2 = 10.0–19.99%, 3 = 20.0–29.9%, 4 = 30.0–49.99%, and 5 = >50.0%. Similarly, IgM seroprevalence was categorized as follows: 1 = <0.50%, 2 = 0.5–0.99%, 3 = 1.0–1.99%, 4 = 2.0–4.99%, 5 = >5.0%.

### Statistical analysis

Prior to the main analyses, we log-transformed data that were not normally distributed to improve normality. Pearson’s correlation coefficients were calculated to evaluate bivariate associations among the variables. For an exploratory analysis, a linear regression model was used to examine the influence of cat density, health expenditure, GDP, income, and *T. gondii* seroprevalence, included as independent variables, on national happiness scores, included as the dependent variable. Based on the regression results, a mediation analysis (5000 bootstrap samples) was then performed introducing IgM seroprevalence as the mediator of the association between cat density and national happiness. As this is an exploratory study, we reported unadjusted *p*-values and focused on effect sizes over statistical significance to minimize the risk of overlooking potentially meaningful associations [53,54]. All analyses were performed using Jamovi (version 2.3.28, [55]) and JASP (version 0.95.3.0, [56]). All tests were two-tailed and statistical significance was set at *p* ≤ 0.05.

It is important to emphasize that since the dataset presented in this study comprises a finite number of countries chosen based on data availability rather than random sampling, our inferential statistics (e.g., *p* values) are interpreted here as indicators of model-based and design-based uncertainty, rather than as sampling-based inferences to a larger population [57,58]. This approach aligns with recent research using hypothesis-testing to assess the robustness and internal consistency of observed associations in cross-national and ecological analyses [53,57].

## Results

### Descriptive analysis

Descriptive data for all variables are shown in Table 2. The study variables did not satisfy the assumption of normality and therefore were log-transformed.

**Table 2:** Descriptive statistics of the study variables.

Variables	Mean (SD)	95%CI	Minimum	Maximum	Shapiro-Wilk	
					*W*	*p*
IgM	2.88 (1.284)	2.62-3.15	1	5	0.904	< 0.001
IgG	3.33 (1.210)	3.08-3.58	1	5	0.882	< 0.001
GDP	24,047.68 (27,022.096)	18,482.55-29,612.82	415.70	128,678.20	0.803	< 0.001
Income group	3.14 (0.904)	2.95-3.33	1	4	0.802	< 0.001
Health expenditure	7.82 (3.306)	7.14-8.50	2.36	21.83	0.937	< 0.001
Cat density	989.28 (769.516)	830.80-1,147.76	67.35	3,245.80	0.915	< 0.001
Happiness	5.72 (1.118)	5.48-5.97	1.72	7.74	0.956	0.003

*Note*: GDP = Gross Domestic Product (in current $US Dollars), IgM and IgG = Immunoglobulin M and G, respectively.

### Correlations

Correlation analyses using both Spearman’s rho and Pearson’s *r* yielded consistent outcomes and revealed multiple associations between the variables. Here, we report the parametric results (see Fig 2). Full results for both methods are summarized in S1 Table in Supplementary Materials. In brief, national happiness scores showed moderate-to-large and positive correlations with cat density (*r* = 0.620, *p* < 0.001, 95%CI = 0.480, 0.733), health expenditure (*r* = 0.350, *p* < 0.001, 95%CI = 0.162, 0.521), income level (*r* = 0.780, *p* < 0.001, 95%CI = 0.686, 0.849), and GDP (*r* = 0.800, *p* < 0.001, 95%CI = 0.718, 0.866). In contrast, happiness scores showed moderate-to-large and negative correlations with *T. gondii* IgM seroprevalence (*r* = −0.540, *p* < 0.001, 95%CI = −0.672, −0.380), and IgG seroprevalence (*r* = −0.330, *p* = 0.001, 95%CI = −0.504, −0.141). Cat density was largely and positively correlated with income (*r* = 0.770, *p* < 0.001, 95%CI = 0.676, 0.844), GDP (*r* = 0.790, *p* < 0.001, 95%CI = 0.699, 0.856), and health expenditure (*r* = 0.600, *p* < 0.001, 95%CI = 0.448, 0.714). A small and negative correlation between cat density and IgG seroprevalence was also observed (*r* = −0.200, *p* = 0.058, 95%CI = −0.385, 0.007). Income level and GDP showed a large and positive correlation (*r* = 0.930, *p* < 0.001, 95%CI = 0.895, 0.953). Income level was largely and negatively correlated with IgM seroprevalence (*r* = −0.530, *p* < 0.001, 95%CI = −0.661, −0.364) and moderately and negatively with IgG seroprevalence (*r* = −0.320, *p* = 0.002, 95%CI = −0.493, −0.126). GDP showed a large and negative correlation with IgM seroprevalence (*r* = −0.590, *p* < 0.001, 95% CI = −0.710, −0.442) and a moderate and negative correlation with IgG seroprevalence (*r* = −0.330, *p* = 0.001, 95%CI = −0.502, −0.138). Both income level and GDP were largely and positively correlated with happiness, cat density, and health expenditure (*r* > 0.570, *p* < 0.001, in all cases). IgM and IgG seroprevalence were moderately and positively correlated (*r* = 0.460, *p* < 0.001, 95%CI = 0.282, 0.606), and both were moderately-to-largely and negatively correlated with socioeconomic indicators and happiness levels (*r* < −0.182, *p* < 0.076, in all cases). These findings support our hypothesis that higher levels of wealth, public investment, and cat density are associated with lower *T. gondii* exposure and greater national happiness. See S1 and S2 Figs in Supplementary Materials for an exploratory income group-stratified analysis.

**Fig 2:**
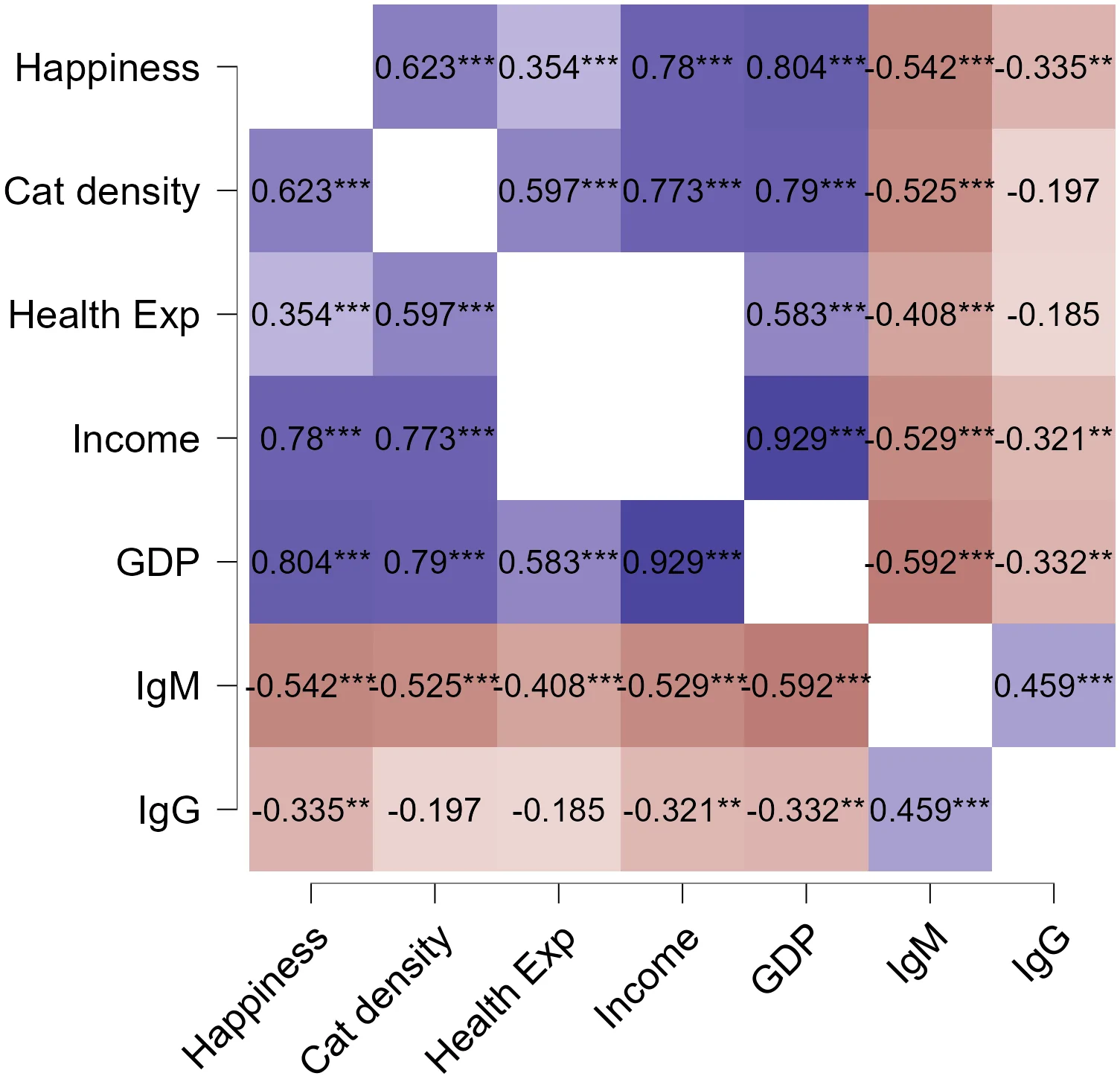
Heatmap showing the correlation between the study variables. Darker shades represent stronger correlations, with purple indicating positive and red indicating negative relationships. *Note*: Health Exp = Health expenditure; GDP = Gross Domestic Product per capita; IgM = Immunoglobulin M; IgG = Immunoglobulin G.

### Exploratory prediction and mediation analyses

1. Due to high multicollinearity between GDP and income (VIFs = 7.61 and 7.41, respectively) these variables were excluded from the exploratory regression analysis. While residuals showed a non-normality distribution (Shapiro-Wilk *p* < 0.05), the model was robust to this deviation given the sample size (*N* = 93) and the maintenance of linearity and homoscedasticity. Health expenditure was retained as the primary socioeconomic indicator, as it represents a more direct proxy for national wealth (for example, because it relates to public health infrastructure). Therefore, only cat density, health expenditure, and *T. gondii* IgM and IgG seroprevalence were included as predictors (VIF < 1.71 in all cases). Our model accounted for 47.0% of the variance in national happiness (*R*^*2*^ = 0.470, *F*_(4,88)_ = 19.500, *p* < 0.001) and showed that cat density positively (*β* = 0.520, *p* < 0.001) and IgM negatively (*β* = −0.240, *p* = 0.021) significantly contributed to the prediction of happiness scores. Small and non-significant associations were found for health expenditure and IgG seroprevalence and their relationship with happiness (Table 3). We also conducted the regression analyses treating IgM and IgG seroprevalence as categorical predictors using dummy coding instead of continuous predictors. The pattern of results remained qualitatively identical (S2 Table in Supplementary Materials), indicating that the reported associations were not driven by the assumption of equal spacing between seroprevalence categories. The exploratory mediation analysis (Fig 3) showed that cat density had a positive direct effect on national happiness, and that this effect was negatively mediated by IgM seroprevalence (Table 4). Overall, our findings consistently showed the strength and internal consistency of the associations between national happiness, cat density, *T. gondii* exposure and socioeconomic factors. Here the mediation analyses are intended as a descriptive statistical decomposition of associations among variables and do not imply causal ordering or mechanistic pathways.

**Table 3:** Results from the regression analysis.

Variables	*b*	SE	*t*	*p*	95%CI
Constant	0.559	0.074	7.506	< 0.001	0.411, 0.707
Health Expenditure	−0.042	0.054	−0.784	0.435	−0.150, 0.065
Cat density	0.114	0.023	4.905	< 0.001**	0.068, 0.161
IgM	−0.019	0.008	−2.358	0.021*	−0.037, −0.003
IgG	−0.012	0.007	−1.567	0.121	−0.027, 0.003

*Note*: IgM and IgG = Immunoglobulin M and G, respectively.

**Table 4:** Path estimates of the mediation analysis.

				95% Confidence Interval			
			Estimate	Lower	Upper	*Z*	*p*
Cat density	→	IgM	−1.4060	−1.8466	−0.9765	−6.29	<.001
IgM	→	Happiness	−0.0247	−0.0389	−0.0112	−3.52	<.001
Cat density	→	Happiness	0.1040	0.0549	0.1616	3.87	<.001

*Note*: IgM = Immunoglobulin M.

**Fig 3:**
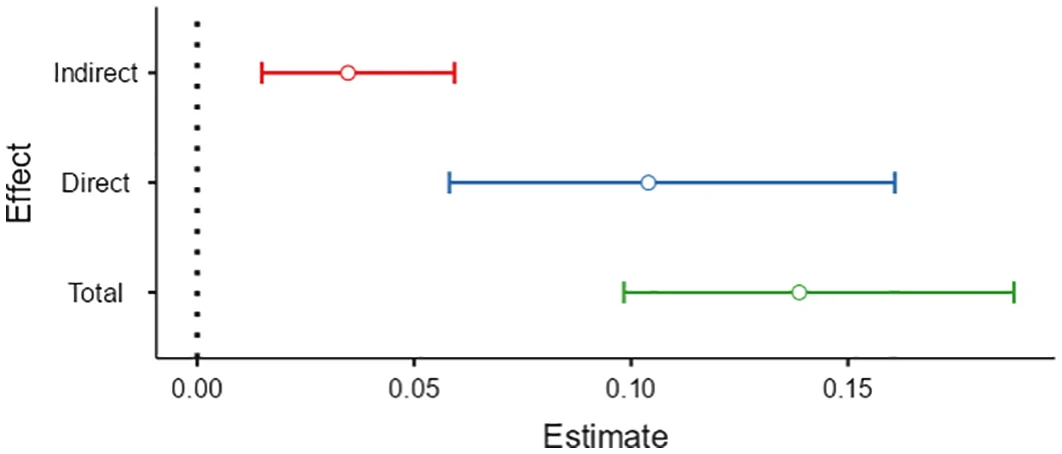
Effects of the mediation analysis representing a statistical decomposition of association between cat density, IgM seroprevalence, and national happiness: Indirect effect (*β* = 0.034, SE = 0.011, 95%BootCIs [0.015, 0.059], *p* = 0.002), Direct effect (*β* = 0.104, SE = 0.026, 95%BootCIs [0.055, 0.161], *p* < 0.001), Total effect (*β* = 0.139, SE = 0.023, 95%BootCIs [0.096, 0.188], *p* < 0.001).

## Discussion

Consistent with our hypotheses, our findings showed that cat density and some socioeconomic indicators were moderately-to-largely and positively associated with happiness, whereas *T. gondii* IgM and IgG seroprevalence were moderately-to-largely and negatively associated with happiness. These results underscore a complex and yet understudied relationship between human-animal interactions, infectious disease ecology, and national well-being.

The observed positive association between cat density and happiness is consistent with previous studies showing the psychological and physiological benefits of pet ownership [e.g., 18–21], including reduced loneliness and improved emotional well-being [16,17,59]. Cat density is an aggregate measure and, therefore, an imperfect proxy for pet ownership and does not capture the quality or context of human-cat interactions. Nevertheless, these associations suggest that human-animal relationships may explain population-level differences in subjective well-being, even when examined at a coarse ecological scale. Thus, it is possible that the links between cat ownership, reduced loneliness, and alleviated stress may explain the association between cat density and greater national happiness, which, in turn, may reflect more favorable attitudes toward pets, higher disposable income, and better living conditions, all of which are closely related to higher subjective well-being [1,3,59].

An interesting and counterintuitive finding of this study was the moderate-to-large negative association between cat density and *T. gondii* seroprevalence, challenging the common assumption that a higher number of cats would imply more parasite exposure [e.g., 46]. One explanation for this finding is that in wealthier countries, cats are more likely to be kept indoors, fed processed food, and receive routine medical care, significantly reducing the potential of *T. gondii* infection. In contrast, in lower- and middle-income countries, cats are often free-roaming, increasing the odds of *T. gondii* infection [27,44,47].

It is noteworthy that while causality cannot be inferred from these ecological correlations, the moderate-to-large associations further support the growing evidence linking *T. gondii* exposure to negative neuropsychological outcomes [33,60]. For instance, chronic *T. gondii* infection has been associated with subtle changes in personality and the development of depression, anxiety, schizophrenia, and other mental disorders [23–25,29,30,35–37,40–42], whereas acute infections may result in immunological alterations that could affect mood and cognitive functioning [25,39]. It is possible that the effects of *T. gondii* infection on psychological processes are mediated by the action of this neuroparasite on brain neurotransmitters and specific circuits involved in emotion regulation and cognitive processes [61,62].

It is important to emphasize that all associations reported here, including those derived from mediation models, should be interpreted as statistical relationships rather than evidence for causal pathways. While ecological correlations can be informative for identifying broad-scale patterns and generating hypotheses, they cannot disentangle directionality or rule out unmeasured confounding processes [61,62]. Accordingly, mediation effects are discussed cautiously and are framed as indirect statistical associations rather than causal effects. It also is critical to highlight that our findings describe associations at the national level and should not be used to infer individual-level effects or personal risks related to cat ownership, happiness, and *T. gondii* exposure.

From a broader and more general perspective, the large associations between GDP, income level, health expenditure, and happiness support the social determinants of health framework [63], according to which healthcare, education, and environmental safety are known to reduce the exposure to infectious agents and enhance psychological resilience [64,65]. Our findings extend this framework by suggesting that cat ownership and parasitic exposure, which is typically overlooked in well-being research, can influence well-being at the national level. The results of our exploratory regression and mediation analyses further clarify the associations that emerged from the correlational analyses. Specifically, these analyses showed that cat density and IgM seroprevalence have the largest associations with national happiness, suggesting that biological and ecological factors may contribute to national well-being alongside established socioeconomic drivers. Additionally, our findings have the potential of informing public health policies aimed to mitigate the negative effects of *T. gondii* exposure, to promote responsible pet ownership, and to increase the well-being at a national level.

### Limitations and strengths

It is important to recognize that our study has several limitations. First, the seroprevalence data on *T. gondii* infection obtained from pregnant women, although widely adopted in epidemiological and ecological research [e.g., 27,28], may not fully represent the broader population introducing a potential demographic bias in our study. It is possible that pregnant women may differ in their healthcare access, testing protocols, and exposure risks across countries. Nevertheless, in the absence of comprehensive national surveillance programs, these data represent the most robust and standardized source for global comparisons. Likewise, the cat density measurement conflates pet cats, stray cats, and feral cat populations which could differently impact on human psychological benefits and parasite transmission processes. Thus, future studies should differentiate between these cat populations and account for cultural variations to more precisely isolate their specific impacts on *T. gondii* exposure and national happiness. Second, we did not consider several confounding variables such as urbanization levels, climate indicators, or sanitation infrastructure, which may also influence cat ownership, parasite exposure, and subjective well-being. For example, it is possible that stray cats are less common in urbanized territories with better sanitation infrastructure, thus limiting the transmission of *T. gondii* to other animals [66]. Moreover, climate indicators such as humidity and temperature may affect the sporulation of oocysts [67] which may also influence processes linked to *T. gondii* prevalence, population health, cat ownership, and ultimately, national well-being.

Third, the cross-sectional ecological data and the correlational design of this study prevented us from inferring causality. Thus, the observed patterns in our exploratory prediction and mediation analyses should be interpreted as a statistical decomposition of associations. Alternative explanations, including reverse causation and unmeasured confounding variables cannot be ruled out and may account for some of the observed relationships. Future studies may benefit from using a predictive approach, rather than a correlational one, to test the causal effects of cat density, *T. gondii* exposure, and socioeconomic indicators of wealth on national happiness. Moreover, because the World Happiness Index partly incorporates socioeconomic indicators such as GDP, some degree of conceptual overlap exists between the outcome measure and certain predictors.

Finally, data quality and availability varied across countries, which is common in cross-national ecological studies relying on secondary data, potentially introducing a temporal misalignment among the study variables and limiting the generalizability of the findings. Although this mismatch may introduce some noise or bias, *T. gondii* seroprevalence tends to change slowly over time in the absence of major public health interventions. Thus, any potential bias is likely conservative, as declining exposure in wealthier countries over time would probably underestimate the negative association between *T. gondii* and national happiness. Future studies should closely align temporal data to confirm the robustness of these ecological patterns.

Despite these limitations, our study also has several strengths. First, while previous research has explored the potential role of parasites such as *T. gondii* on economic and cultural patterns [e.g., 43,68], this is the first study that integrates ecological, biological, and socioeconomic indicators in a global analysis of happiness across a large set of countries. By including data on cat ownership, parasitic exposure, and economic development, this research provides a novel and interdisciplinary perspective on the determinants of well-being. Moreover, the inclusion of both IgM and IgG seroprevalence allowed us to differentiate between acute and chronic infections, increasing the biological specificity of our analysis. Overall, our findings highlight the importance of considering both biological and socio-ecological factors in the study and understanding of global patterns of subjective well-being.

## Conclusion

This ecological study provides novel insights into the magnitude and robustness of the associations between happiness, cat density, *T. gondii* exposure, and socioeconomic factors across 93 countries. Our main findings showed that, besides the typical associations between subjective well-being and some socioeconomic factors, national happiness was largely and positively associated with cat density and largely and negatively associated with IgM *T. gondii* exposure. These results underscore the need for future studies integrating both socioeconomic and biological determinants of happiness and including longitudinal and interdisciplinary approaches to elucidate the underlying mechanisms and processes.

## Supporting information

S1 Fig Cat density and national happiness. Correlation between cat density and national happiness stratified by income level. *Note:* Income groups: green = low, gray = lower-middle, purple = upper-middle, yellow = high.
(DOCX)

S2 Fig IgM and national happiness. Correlation between IgM seroprevalence and national happiness stratified by income level. *Note:* IgM = Immunoglobulin M; Income groups: green = low, gray = lower-middle, purple = upper-middle, yellow = high.
(DOCX)

S1 Table Pearson’s *r* and Spearman rho correlations. Correlations among the study variables.
(DOCX)

S2 Table Regression analysis. Regression analysis including IgM and IgG seroprevalence as categorical predictors using dummy coding.
(DOCX)

S1 File Dataset. Dataset used in this study.
(XLSX)
