# Supplementary material for: Wealth, health, cats and Toxoplasma gondii: An ecological correlational study of subjective well-being across 93 countries
Source: PLoS One. 2026 Sep 30;21(9):e0357911. doi: 10.1371/journal.pone.0357911 (PMC13626322; doi:10.1371/journal.pone.0357911)
Supplement: Supplementary file 2 [file pone.0357911.s002.docx]

**Figure S2**. Correlation between IgM seroprevalence and national happiness stratified by income level. *Note:* Income groups: green = low, gray = lower-middle, purple = upper-middle, yellow = high.
