# Supplementary material for: Wealth, health, cats and Toxoplasma gondii: An ecological correlational study of subjective well-being across 93 countries
Source: PLoS One. 2026 Sep 30;21(9):e0357911. doi: 10.1371/journal.pone.0357911 (PMC13626322; doi:10.1371/journal.pone.0357911)
Supplement: Supplementary file 3 [file pone.0357911.s003.docx]

| **Table S1**. Correlations among the study variables. | | | | | | | | | | | | | |
| --- | --- | --- | --- | --- | --- | --- | --- | --- | --- | --- | --- | --- | --- |
|  | | | | | | Pearson | | | | Spearman | | | |
|  | |  | |  | | r | | p | | rho | | p | |
| Happiness |  | - |  | Cat density |  | 0.623 | *** | < .001 |  | 0.670 | *** | < .001 |  |
| Happiness |  | - |  | Health Exp |  | 0.354 | *** | < .001 |  | 0.575 | *** | < .001 |  |
| Happiness |  | - |  | Income |  | 0.780 | *** | < .001 |  | 0.827 | *** | < .001 |  |
| Happiness |  | - |  | GDP |  | 0.804 | *** | < .001 |  | 0.899 | *** | < .001 |  |
| Happiness |  | - |  | IgM |  | -0.542 | *** | < .001 |  | -0.599 | *** | < .001 |  |
| Happiness |  | - |  | IgG |  | -0.335 | ** | .001 |  | -0.387 | *** | < .001 |  |
| Cat density |  | - |  | Health Exp |  | 0.597 | *** | < .001 |  | 0.630 | *** | < .001 |  |
| Cat density |  | - |  | Income |  | 0.773 | *** | < .001 |  | 0.752 | *** | < .001 |  |
| Cat density |  | - |  | GDP |  | 0.790 | *** | < .001 |  | 0.772 | *** | < .001 |  |
| Cat density |  | - |  | IgM |  | -0.525 | *** | < .001 |  | -0.530 | *** | < .001 |  |
| Cat density |  | - |  | IgG |  | -0.197 |  | .058 |  | -0.217 | * | .037 |  |
| Health Exp |  | - |  | Income |  | 0.569 | *** | < .001 |  | 0.601 | *** | < .001 |  |
| Health Exp |  | - |  | GDP |  | 0.583 | *** | < .001 |  | 0.648 | *** | < .001 |  |
| Health Exp |  | - |  | IgM |  | -0.408 | *** | < .001 |  | -0.429 | *** | < .001 |  |
| Health Exp |  | - |  | IgG |  | -0.185 |  | .076 |  | -0.183 |  | .079 |  |
| Income |  | - |  | GDP |  | 0.929 | *** | < .001 |  | 0.928 | *** | < .001 |  |
| Income |  | - |  | IgM |  | -0.529 | *** | < .001 |  | -0.536 | *** | < .001 |  |
| Income |  | - |  | IgG |  | -0.321 | ** | .002 |  | -0.355 | *** | < .001 |  |
| GDP |  | - |  | IgM |  | -0.592 | *** | < .001 |  | -0.614 | *** | < .001 |  |
| GDP |  | - |  | IgG |  | -0.332 | ** | .001 |  | -0.334 | ** | .001 |  |
| IgM |  | - |  | IgG |  | 0.459 | *** | < .001 |  | 0.462 | *** | < .001 |  |
|  | | | | | | | | | | | | | |
| * p < .05, ** p < .01, *** p < .001 | | | | | | | | | | | | | |
