# Supplementary material for: Wealth, health, cats and Toxoplasma gondii: An ecological correlational study of subjective well-being across 93 countries
Source: PLoS One. 2026 Sep 30;21(9):e0357911. doi: 10.1371/journal.pone.0357911 (PMC13626322; doi:10.1371/journal.pone.0357911)
Supplement: Supplementary file 4 [file pone.0357911.s004.docx]

**Table S2**. Regression analysis including IgM and IgG seroprevalence as categorical predictors using dummy coding.

|  | | | | | | | | | |
| --- | --- | --- | --- | --- | --- | --- | --- | --- | --- |
|  | | | **95% Confidence Interval** | |  | | | **95% Confidence Interval** | |
| **Predictor** | **Estimate** | **SE** | **Lower** | **Upper** | **t** | **p** | **Stand. Estimate** | **Lower** | **Upper** |
| Constante | 0.5590 | 0.07447 | 0.4110 | 0.70701 | 7.506 | <.001 |  |  |  |
| IgM | -0.0199 | 0.00846 | -0.0368 | -0.00314 | -2.358 | 0.021 | -0.2395 | -0.441 | -0.0377 |
| IgG | -0.0121 | 0.00774 | -0.0275 | 0.00326 | -1.567 | 0.121 | -0.1372 | -0.311 | 0.0369 |
| Log10HealthExp | -0.0426 | 0.05435 | -0.1506 | 0.06543 | -0.784 | 0.435 | -0.0766 | -0.271 | 0.1177 |
| Log10Catsx10000 | 0.1149 | 0.02342 | 0.0684 | 0.16145 | 4.905 | <.001 | 0.5156 | 0.307 | 0.7244 |
